# On family-based genome-wide association studies with large pedigrees: observations and recommendations

**DOI:** 10.1186/1753-6561-8-S1-S26

**Published:** 2014-06-17

**Authors:** David W Fardo, Xue Zhang, Lili Ding, Hua He, Brad Kurowski, Eileen S Alexander, Tesfaye B Mersha, Valentina Pilipenko, Leah Kottyan, Kannabiran Nandakumar, Lisa Martin

**Affiliations:** 1 Department of Biostatistics, University of Kentucky College of Public Health, 111 Washington Ave, Lexington, KY 40536, USA; 2 Department of Pediatrics, Cincinnati Children's Hospital Medical Center, 3333 Burnet Avenue, Cincinnati, OH 45229, USA; 3 Department of Pediatrics, University of Cincinnati College of Medicine, 2600 Clifton Ave, Cincinnati, OH 45229, USA; 4 Department of Environmental Health, University of Cincinnati College of Medicine, 2600 Clifton Ave, Cincinnati, OH 45229, USA

## Abstract

Family based association studies are employed less often than case-control designs in the search for disease-predisposing genes. The optimal statistical genetic approach for complex pedigrees is unclear when evaluating both common and rare variants. We examined the empirical power and type I error rates of 2 common approaches, the measured genotype approach and family-based association testing, through simulations from a set of multigenerational pedigrees. Overall, these results suggest that much larger sample sizes will be required for family-based studies and that power was better using MGA compared to FBAT. Taking into account computational time and potential bias, a 2-step strategy is recommended with FBAT followed by MGA.

## Background

Phenotypic variation in complex traits is conferred through both common and rare variants. It has been suggested that common variation plays a role at the level of the population, whereas rare variation has stronger effects at the levels of the clan (extended family) and the nuclear family [1]. To date, a large number of genome-wide association studies (GWAS) have focused on population-level variation. Since the first GWAS was published in 2005 [2], more than 1000 have been conducted. By using predominantly case-control designs with single-variant analyses, these studies have identified common variants associated with common diseases and related phenotypes. Alternatively, family-based approaches using trios and nuclear families have been increasingly utilized with GWAS and next-generation sequencing [3-9]. In the past 10 years, studies of extended families have been much more limited, even though individuals sharing recent ancestors share regions of the genome other than disease-causing variants and may provide a better proxy for the total mutation load [1]. Thus, there is a clear need to evaluate strategies for the analysis of genetic data from extended families.

The measured genotype approach (MGA) and family-based association testing (FBAT) are 2 broad strategies to examine family-based association in the context of large extended families. MGA from a variance components framework utilizes a mixed model in which familial relationships are accounted for using random effects and genetic variants are incorporated as fixed effects. In contrast, FBAT relies solely on within-family information by constructing a score test that essentially provides a correlation between phenotype and genotype. However, performance of these approaches in the context of variants of varying frequency with modest to moderate effect in extended family data is unclear.

Thus, this paper evaluates the performance of MGA and FBAT in the context of large extended families genotyped for both common and rare variants (minor allele frequency ≥5% and <5%, respectively). To accomplish this, we will use chromosome 3 variants from single-nucleotide polymorphism (SNP) genotyping chips, as well as the simulated phenotypes from the Genetic Analysis Workshop 18 (GAW18) data set based on the multigenerational structure of the San Antonio Family Studies (SAFS) [10].

## Methods

We analyze 20 large pedigrees generated from SAFS that range from 21 to 76 members in size. We used the chromosome 3 data to test for association in the 200 simulation replicates by employing both MGA [11] and FBAT [6,12] with diastolic blood pressure (DBP) at exam 1. To assess empirical false-positive rates, we analogously analyze Q1, a trait simulated with no genetic link.

Details regarding the San Antonio Family Heart Study (SAFHS) and the San Antonio Family Diabetes/Gallbladder Study (SAFDGS), which comprise the SAFS, have been provided elsewhere [13,14]. Pertinent to our analyses, GWAS data were generated from this study using a variety of genotyping platforms and extensively cleaned, resulting in a total of 472,049 SNPs. The 65,519 SNPs residing on chromosome 3 were used in our analyses.

### Measured genotype approach

First, we used MGA [9,15] as implemented in SOLAR (Texas Biomedical Research Institute, San Antonio, TX) [16]. This approach accounts for phenotypic correlation between family members by including a polygenic component as a random effect. Each SNP is coded additively (ie, as a count of minor alleles) and is incorporated as a fixed effect in the following model:

(1) $$DBP=\mu +\beta_{1}age+\beta_{2}age^{2}+\beta_{3}BPMED+\beta \times \left(SNP\right)+g+e$$

where *μ *is a grand mean for DBP, $\beta_{1},\beta_{2},\beta_{3}$ are the respective covariate effects, *β *is the SNP effect, and *g *and *e *are random genetic (additive polygenic) and residual effects. We assume that *g *and *e *are normally distributed with zero mean and variances $2\text{Φ}\sigma_{g}^{2}$ and $\text{I}\sigma_{e}^{2}$, respectively, where Φ is the kinship matrix, I is the identity matrix, and ${\sigma_{g}}^{2}$, ${\sigma_{e}}^{2}$ are the variances from additive genetic (*g*) and residual (*e*) effects. To test a SNP effect, the log likelihood of the model estimating an unconstrained SNP effect is compared to the log likelihood of the model in which the SNP effect is constrained to zero. Assuming that trait values follow a multivariate normal distribution, twice the difference in the log likelihoods of these 2 models is asymptotically distributed as $\chi_{1}^{2}$.

### Family based association test: marginal tests

Second, we used FBAT to test for association. Here we define the FBAT test statistic by

(2) $$\underset{ij}{\sum }\frac{t_{ij}\left(x_{ij}-E\left(x_{ij}|S_{ij}\right)\right)}{t_{ij}^{2}Var\left(x_{ij}|S_{ij}\right)}\sim \chi_{1}^{2}$$

where $t_{ij}$ is residual phenotype (DBP at exam 1) from the *j*th nonfounder of the *i*th family after regression on age, age squared, sex, and blood pressure medication use, all at the first exam; $x_{ij}$ is the additively coded genotype (ie, minor allele count) for this subject; and $S_{ij}$ are the sufficient statistics [17] for the *j*th nonfounder of the *i*th family (eg, the sufficient statistics consist of parental genotypes when analyzing mother-father-offspring trios). FBAT analysis was performed with PBAT's [18] hybrid pedigree algorithm that clusters trios within extended pedigrees to improve computation time using SNP & Variation Suite v7.6.10 (Golden Helix, Bozeman, MT, http://www.goldenhelix.com).

### Family based association test: screening approach

In addition to examining FBAT test statistics marginally, we also employed the Van Steen screening approach [19], which allows for a reduction in the multiple comparisons burden. Briefly, the screening method imputes nonfounder variants by conditioning on the corresponding sufficient statistics and then estimates the conditional power for each variant. This metric is then used to screen, or rank, variants for testing, thereby reducing the adjustment necessary to declare statistical significance. Extensions of this have been proposed [20]; here, for simplicity of exposition, we use the simple top 10 approach, as done in Herbert et al [21], of testing only the top 10 variants based on conditional power using a Bonferroni-corrected significance threshold of 0.05/10.

### Power

Each of the 17 SNPs from the simulation model that are causal for DBP $\left(\left|{\hat{\beta }}_{DBP}\right|>0\right)$ was tested with MGA and FBAT using a nominal 5% significance threshold. The Bonferroni correction was calculated slightly differently for MGA and FBAT. For MGA analyses, 62,715 SNPs were considered (monomorphic SNPs were removed), resulting in a 0.05/62715 significance threshold. These same SNPs were examined using FBAT, and only the 58,519 SNPs that included at least 10 informative families were tested, giving a Bonferroni-corrected significance of 0.05/58519.

### Type I error

To assess false-positive rates, we examined the trait Q1 simulated with no genetic influence. Linkage disequilibrium (LD) was used to prune the chromosome 3 SNPs and create a subsample of 1228 uncorrelated SNPs. These SNPs were used to estimate type I error rates, using both MGA and FBAT to maintain consistency across approaches. The pruning approach has 2 advantages. First, it reduces the computational burden, which was especially problematic in MGA where computation time increases substantially with the degree of pedigree complexity as a result of estimation of the mixed model. Second, it results in an error rate more in line with the number of true comparisons, as Bonferroni correction assumes uncorrelated tests. To calculate a comparable assessment of type I error using the Van Steen screening approach, the proportion of noncausal SNPs declared significant in each replicate was averaged.

Of note, the multiple testing correction approach differed between the power and the type I error evaluation. Specifically, the LD pruning step was not performed when examining empirical power. Although it is optimal to use the same procedure to assess error rate and power, the varying pruning step should not bias our results.

## Results

### Power

Overall, there was low power to detect causal variants (Table 1). Only 3 SNPs achieved greater than 20% power using a nominal significance level. SNP rs11711953 in *MAP4 *had a considerably large effect on DBP (heritability 2.29%) and a minor allele frequency (MAF) of 2.6%. The other 2 SNPs with marginal power, rs4683602 and rs16851435, are common (MAFs of 0.272 and 0.243, respectively) but exhibited a much more modest effect (heritability 0.003% and <10^−5^). After accounting for multiple testing, only rs11711953 had the power to be detected, and then only by using MGA. When using the Van Steen top 10 screening approach (FBAT-VS) the *MAP4 *SNP was detectable, but not at the rate conferred by MGA.

**Table 1:** Empirical powers for DBP causal variants.

	Characteristics				No correction		Bonferroni correction		
	
SNP	Gene	MAF	Effect Size	Heritability	MGA	FBAT	MGA	FBAT	FBAT-VS
rs304079	SUMF1	0.4828	0.0895	0.00005	0.015	0.010	0	0	0
rs373572	RAD18	0.3707	0.0002	0	0.050	0.015	0	0	0
rs1800734	MLH1	0.3190	−0.1142	0.00007	0.005	0.060	0	0	0
rs2020873	MLH1	0.0135	−0.4753	0.00005	0.035	0*	0	0*	0*
**rs11711953**	**MAP4**	**0.0261**	−**6.2235**	**0.02290**	**1.000**	**0.310**	**0.995**	**0.000**	**0.370**
rs1131356	FLNB	0.4955	0.3875	0.00085	0.180	0.090	0	0	0
rs3772985	DNASE1L3	0.1983	−0.0795	0.00003	0.015	0.015	0	0	0
rs12491947	DNASE1L3	0.0766	0.0005	0	0.020	0	0	0	0
rs9815775	DNASE1L3	0.3103	0.037	0.00001	0.015	0.060	0	0	0
rs2322142	PROK2	0.4234	−0.0678	0.00003	0.015	0.015	0	0	0
rs6438503	B4GALT4	0.1595	−0.1248	0.00004	0.020	0.025	0	0	0
rs6805930	B4GALT4	0.0496	0.1855	0.00004	0.055	0.005	0	0	0
rs4679394	MUC13	0.1897	−0.0891	0.00003	0.035	0.015	0	0	0
rs9814557	PPP2R3A	0.1293	0.0057	0	0.020	0.005	0	0	0
rs9826032	PPP2R3A	0.0135	0.0006	0	0.055	0*	0	0*	0*
**rs4683602**	**ZBTB38**	**0.2716**	**0.0725**	**0.00003**	**0.220**	**0.105**	**0**	**0**	**0**
**rs16851435**	**ZBTB38**	**0.2432**	−**0.0041**	**0**	**0.405**	**0.140**	**0**	**0**	**0**

Results from 200 Genetic Analysis Workshop (GAW) simulations for MGA, FBAT and FBAT-VS (the FBAT top 10 screening approach). SNPs conferring at least 20% power for any method are indicated in bold. The gene, minor allele frequency (MAF; estimated from founders), effect size, and heritability are provided. Results without multiple testing correction are listed under "No correction." Methods with a genome-wide correction are under "Bonferroni correction." Entries marked with an asterisk (*) were not tested with FBAT methods because of a lack of informative families.

### Type I error

Using the Q1 phenotype, we found that both MGA and FBAT methods appropriately controlled for type I error rate using a nominal significance (type I error rate 0.05 for both). After controlling for multiple testing, no false positives were identified with any of the methods.

## Discussion and conclusions

Using a cohort of extended families, we evaluated the performance of 2 family based methods (MGA and FBAT) to identify causal variants of varying allele frequency and effect size. Overall, the approaches exhibited low power with only 3 variants identified more than 20% of the time. Nevertheless, both approaches also exhibited very appropriate family-wise false-positive rates. Taken together, these results suggest that family-based studies require large sample sizes to detect the majority of effects.

The variant identified across all approaches (rs11711953), had a MAF of 0.026 and a true effect size of −6.2235 (with heritability of 2.29%). It appears that the ability to detect this variant was driven by the very strong effect size (more than 10× greater than any other variant). The other 2 variants identified were more common, but had relatively small effect sizes. As other common variants had larger effect sizes, there is clearly a complex interplay of factors influencing power to detect effects.

Both methods suffered from overall low power. This suggests that substantially larger data sets and methodological extensions incorporating multiple variants such as FBAT-RV [22] will be required when testing for effects of rare variants on complex phenotypes. However, care is required to prevent spurious association results when increasing sample size. Specifically, because the measured genotype approach is susceptible to confounding as a result of population stratification, combining data across multiple studies may be problematic. In the current study, there were no inflated false-positive rates using any of the methodologies, suggesting that there were no adverse effects of population stratification. However, given the extreme low power of this study, care must be taken to not overevaluate these findings. Future studies need to explore this possibility with more genetically diverse family samples to examine the relative merits of family-based approaches. Notably, methods that rely on between-family information must appropriately handle population stratification because their validity is contingent on either its absence [23] or sufficient adjustment, as opposed to FBAT approaches that are, by design, robust to population stratification.

One of the major challenges in these analyses was the computational time, especially for the MGA, where genome-wide analyses are infeasible. MGA analysis took approximately 30 seconds per SNP, while the FBAT took one-eighth second per SNP. Ideally, without any constraints on computation time and with sufficient evidence to rule out population stratification, it is best to perform both MGA and FBAT approaches across the genome and focus on regions of overlap, that is, those with most evidence for true association. However, because both time and population substructure are often constraints, when considering between MGA- or FBAT-type analyses, we recommend initially employing an FBAT screening approach with a less-stringent significance threshold because of its speed and robustness to population stratification, and then following up regions of interest with MGA for confirmation to identify variants most likely to be causal.

In summary, analysis of the GAW18 simulated phenotypes, DBP and Q1, allowed us to examine the performance of family-based association methods in the context of extended families and variants of varying frequency. Overall, we found that the GAW18 data was underpowered to detect all but one of the variants regardless of the approach used. Approaches to ease the burden of multiple testing are beneficial, and simulations with explicit population stratification are needed to further discern comparisons between these methods.

## Competing interests

The authors declare that they have no competing interests.

## Authors' contributions

DWF, XZ, and LJM designed the overall study. DWF, XZ, and KN conducted the statistical analyses and created tables. DWF, XZ, and LJM drafted the manuscript, which was revised by LD, HH, BGK, ESA, TBM, VP, LK, and KN. All authors discussed the project throughout, read, and approved the final manuscript.
